# Incidence, healthcare-seeking behaviours, antibiotic use and natural history of common infection syndromes in England: results from the Bug Watch community cohort study

**DOI:** 10.1186/s12879-021-05811-7

**Published:** 2021-01-22

**Authors:** Catherine M. Smith, Laura J. Shallcross, Peter Dutey-Magni, Anne Conolly, Christopher Fuller, Suzanne Hill, Arnoupe Jhass, Franziska Marcheselli, Susan Michie, Jennifer S. Mindell, Matthew J. Ridd, Georgios Tsakos, Andrew C. Hayward, Ellen B. Fragaszy, on behalf of the PASS research group, Niall Anderson, Lou Atkins, Anne Conolly, Elise Crayton, Spiros Denaxas, Peter Dutey-Magni, Nadia Elsay, Gillian Forbes, Ellen Fragaszy, Nick Freemantle, Christopher Fuller, Martin Gill, Andrew C. Hayward, Rob Horne, Arnoupe Jhass, Patty Kostkova, Fabiana Lorencatto, Susan Michie, Jennifer S. Mindell, Michelle Richardson, John Robson, Patrick Rockenschaub, Claire Royston, Laura J. Shallcross, Catherine M. Smith, Elizabeth Sutton, James Thomas, Carolyn Tarrant, Rosanna Traina, Emma Richardson, Jonathan West, Haydn Williams

**Affiliations:** 1 Institute of Health informatics, UCL, 222 Euston Road, London, NW1 2DA UK; 2 NatCen Social Research, 35 Northampton Square, London, EC1V 0AX UK; 3 Research Department of Primary Care and Population Health, UCL, Rowland Hill Street, London, NW3 2PF UK; 4 Centre for Behaviour Change, UCL, 1-19 Torrington Place, London, WC1E 7HB UK; 5 Research Department of Epidemiology and Public Health, UCL, 1-19 Torrington Place, London, WC1E 7HB UK; 6 Health Science Institute, University of Bristol, 39 Whatley Road, Bristol, BS8 2PS UK; 7 Institute of Epidemiology and Health Care, UCL, 1-19 Torrington Place, London, WC1E 7HB UK; 8 Faculty of Epidemiology and Population Health, London School of Hygiene and Tropical Medicine, Keppel Street, London, WC1E 7HT UK

**Keywords:** Antibiotic stewardship, common infections, Incidence, healthcare-seeking behaviour, community cohort studies

## Abstract

**Background:**

Better information on the typical course and management of acute common infections in the community could inform antibiotic stewardship campaigns. We aimed to investigate the incidence, management, and natural history of a range of infection syndromes (respiratory, gastrointestinal, mouth/dental, skin/soft tissue, urinary tract, and eye).

**Methods:**

Bug Watch was an online prospective community cohort study of the general population in England (2018–2019) with weekly symptom reporting for 6 months. We combined symptom reports into infection syndromes, calculated incidence rates, described the proportion leading to healthcare-seeking behaviours and antibiotic use, and estimated duration and severity.

**Results:**

The cohort comprised 873 individuals with 23,111 person-weeks follow-up. The mean age was 54 years and 528 (60%) were female. We identified 1422 infection syndromes, comprising 40,590 symptom reports. The incidence of respiratory tract infection syndromes was two per person year; for all other categories it was less than one. 194/1422 (14%) syndromes led to GP (or dentist) consultation and 136/1422 (10%) to antibiotic use. Symptoms usually resolved within a week and the third day was the most severe.

**Conclusions:**

Most people reported managing their symptoms without medical consultation. Interventions encouraging safe self-management across a range of acute infection syndromes could decrease pressure on primary healthcare services and support targets for reducing antibiotic prescribing.

**Supplementary Information:**

The online version contains supplementary material available at 10.1186/s12879-021-05811-7.

## Background

Antimicrobial resistance (AMR) is a serious global problem that has resulted in increasing numbers of infections becoming untreatable [1]. There were over 60,000 severe antibiotic resistant infections in England in 2018; approximately 165 new antibiotic resistant infections per day [2]. As AMR is largely driven by selective pressure exerted through antibiotic use [1], ensuring that antibiotics are used responsibly (antibiotic stewardship) is a priority [3]. Antibiotic prescribing in England reduced by 9% from 2014 to 2018, but the number of drug-resistant infections continues to rise [2]. The UK’s national action plan on tackling AMR aims to further reduce prescribing by 15% by 2024 whilst reducing the number of drug-resistant infections [4].

Achieving this aim requires identification of opportunities to safely reduce prescribing. The majority of antibiotic prescribing in England is in general practice, followed by hospitals, other community settings, and dental practice [2]. Most prescriptions made in general practice are for infections of the respiratory tract, urogenital tract, and skin/wounds [5]. It has been estimated that up to 23% of prescriptions in general practice, and over half of those in dental practice, are inappropriate [6, 7]. This includes prescriptions for mild illnesses that would get better on their own and has led to initiatives to reduce inappropriate prescribing in primary care, and efforts to understand levels of knowledge of antibiotic stewardship amongst professionals and the general public [8, 9]. Encouraging patients to manage their symptoms without medical consultation could be an effective complementary approach to reducing antibiotic prescribing.

There is evidence from large-scale community cohort studies in England that most people can safely self-manage respiratory and gastrointestinal infections without visiting a GP: The Flu Watch study estimated that 21% of influenza-like illnesses led to a GP consultation [10], and the IID2 study found approximately one GP consultation for every 15 cases of infectious intestinal disease in the community [11]. This abundance of undetected community cases is referred to as a clinical “iceberg” of infection and has been used as a evidence for promoting safe self-management of these symptoms [12]. However, there is limited information on how people manage symptoms of other common infections as similar large-scale studies have not been conducted.

Information on the usual duration of symptoms has also been used to develop educational resources for antibiotic stewardship campaigns. For example, Public Health England’s *Keep Antibiotics Working* campaign lists how long most people take to recover from five common illnesses (cough, cold, ear ache, sore throat, and sinusitis) with guidance on what to do if symptoms persist [13]. Establishing these estimates for a wider range of infections requires information on the typical duration of symptoms in the community (i.e., including episodes of symptoms that do not lead to healthcare consultation). Measuring the impacts of infections on quality of life can also inform health economic assessments of potential interventions. Data from the Flu Watch study has been used to generate these estimates for influenza-like illnesses [14] but there is a lack of similar information for other infections.

Bug Watch was a prospective community cohort study in England in 2018–2019 that collected information on symptoms of a range of acute common infections (respiratory, gastrointestinal, mouth/dental, skin/soft tissue, urinary tract, and eye) [15]. We quantified the incidence of these common infection syndromes in the community, determined the proportions of syndromes that led to various healthcare-seeking behaviours and antibiotic use, and estimated the average duration of symptoms and their impacts on health-related quality of life.

## Methods

### Study design, recruitment and data collection

This was an online prospective community cohort study in England. Full details of the study design are described in the protocol [15]. Briefly, we recruited participants through the Health Survey for England (HSE), an annual survey designed to be representative of the population living in private households in England [16]. We sent invitation letters to all adults who had taken part in HSE in 2013, 2014 or 2015 and had consented to be contacted about future research (87% participants). Parents or guardians were asked to register their children aged under 16 (and completed all surveys on their behalf), and any other adults living in the household were invited to register separately. Recruitment was conducted in four waves in March, June, September and November 2018.

Study registration consisted of an online consent form and baseline survey. Baseline data included contact details, demographics, information on general health, number of GP consultations in the last year, health-related quality of life (adults only, measured using the EQ-5D-3L instrument [17]) and questions on knowledge and attitudes towards antibiotics (adapted from the 2015 Wellcome Trust Monitor survey) [18].

During follow-up, participants were asked to keep track of symptoms of infection prospectively using a laminated symptom diary which we sent to them after registration. This included 44 symptoms of infection in seven categories: Non-specific, respiratory, gastrointestinal, mouth/dental, skin/soft tissue, urinary tract, and eye. Each week, participants were then emailed a link to an online survey to report which symptoms (if any) they had on each day. If they had no symptoms in a particular week, they reported this through a very short survey. They were also asked to report associated healthcare-seeking behaviours (GP consultation in person or by telephone, nurse at GP practice, going to hospital, going to a walk-in centre, using an internet doctor, using the NHS 111 service (a free, 24 h non-emergency medical helpline), visiting a pharmacy, looking for information on the internet, or going to the dentist), health-related quality of life (adults only, using EQ-5D-3L), and antibiotic use. Participants were followed-up for 6 months.

Data were collected using Research Electronic Data Capture (REDCap) [19] surveys hosted on the UCL Data Safe Haven, which is certified to the ISO27001 information security standard and conforms to NHS Digital’s Information Governance Toolkit. This study was given ethical approval by the UCL Research Ethics Committee (ID 11813/001).

### Statistical analysis

We calculated the overall proportion of people who responded to the survey invitation and the proportion of weekly surveys completed by each individual. Participants who completed fewer than 75% of weekly surveys were excluded from the analysis. We described the baseline characteristics of the cohort and compared them with those who signed up but were excluded. We also assessed population representativeness by comparing, where relevant, with census population estimates, HSE data, and results from the Wellcome Trust Monitor survey [18].

In our population of participants who completed at least 75% of surveys, we identified infection syndromes (i.e. combinations of symptoms associated with one potential infection episode) by combining reports of specific and non-specific symptoms across different days. A new infection syndrome in a given category was defined when a symptom was reported for the first time or more than 10 days after a previous syndrome (Figure S1). A 10 day cut-off was used for new syndromes because it would allow infection syndromes to span multiple weeks even if one weekly survey was missed. Missing weeks were otherwise assumed to have no symptoms. Non-specific symptoms could contribute to the duration of infection syndromes in any category, but at least one specific symptom had to be reported to define a syndrome in a given category, and non-specific symptoms alone were not classified as infection syndromes. For example, a headache reported in the absence of other infection symptoms was not classified as an infection syndrome as most headaches are not due to infection. Specific symptoms in different categories reported concurrently were classified as separate infection syndromes. We calculated the proportion of infection syndromes in which each symptom was reported.

In calculation of person-time denominators, we excluded the first 10 days of follow-up to remove prevalent infection syndromes. If symptoms were reported in the first 10 days, we started follow-up on the first day with no symptoms. We calculated crude incidence rates for each syndrome per person-year and by month. We also calculated age- and sex-specific and adjusted rates, weighting to the mid-2017 population structure of England [20] using post-stratification (implemented through the R ‘survey’ package [21]).

We calculated the proportion of infection syndromes leading to different healthcare-seeking behaviours and antibiotic use, overall and separately by sex and for adults and children. We described antibiotics used by drug name, source, prescription length, duration taken, whether it was a delayed prescription (i.e. the antibiotic was prescribed but with advice to delay its use in the expectation that symptoms will resolve first), and reasons for not finishing the prescribed course.

To assess the duration of infection syndromes, we excluded syndromes that led to antibiotic use. For these syndromes, we plotted survival curves showing proportions with ongoing symptoms each day. We also summarised the number of consecutive days on which each individual symptom was reported. We estimated the mean and median duration of syndromes by fitting log-logistic models to account for right-censoring of data (further details in Additional File 1).

To assess impacts of infection syndromes on adult health-related quality of life, we converted EQ-5D-3L responses to an index value by mapping to a validated UK data set ranging from one (full health) to zero (dead) [22]. We identified the mean worst day by EQ-5D-3L index value in each syndrome category and estimated the mean quality-adjusted life day (QALD) loss by subtracting daily index values from baseline measurements. We used our estimates of duration of syndromes to calculate the average QALD impact of each type of syndrome (further details Additional File 1).

All analyses and data management were conducted using R version 3·6·1.

## Results

### Recruitment and follow-up

The total number of adults invited was 19,471, and 1063 (5%) signed up. Additional recruitment of other adults (21) and children (158) in the households gave a total of 1242 people registered. Completion of weekly surveys was fairly consistent over the follow-up time (Figure S2). 72% (782/1084) of adults completed 75% or more surveys. Participants included in the analysis therefore comprised these 782 adults and the 91 children that they registered, a total of 873. The total included follow-up time was 23,111 person-weeks (444 person-years).

Baseline characteristics of the cohort are shown in Table 1. The mean age was 54 years; 60% (528/873) of participants were female. Compared with the Wellcome Trust Monitor population, a larger proportion of the Bug Watch cohort knew that antibiotics can only be used to treat bacterial infections (556/782 adults in Bug Watch, 71% vs 41% in Wellcome Trust Monitor) [18]. However, the proportion reporting antibiotic use in the previous year was similar (134/782 adults in Bug Watch, 17% vs 23% in Wellcome Trust Monitor). Full details of the study cohort, those who were excluded, and population comparisons are shown in Additional File 1 (Table S1).

**Table 1:** Baseline cohort characteristics

Variable	N (Total 873)	%
Sex		
Male	343	39·3
Female	528	60·5
Unspecified or prefer not to say	2	0·2
Age group (years)		
5 or less	26	3·0
6–16	65	7·5
17–35	57	6·5
36–55	170	19·5
56–70	377	43·2
71+	174	19·9
Region		
East Midlands	81	9·3
East of England	130	14·9
London	73	8·4
North East	60	6·9
North West	92	10·5
South East	172	19·7
South West	100	11·5
West Midlands	93	10·7
Yorkshire and The Humber	69	7·9
Index of multiple deprivation quintile		
1 (most deprived)	49	5·6
2	155	17·8
3	206	23·6
4	218	25·0
5 (least deprived)	242	27·7
Ethnicity		
White	839	96·1
Black	4	0·5
Asian	12	1·4
Mixed/ multiple ethnic background	10	1·1
Any other ethnic group	7	0·8
Long-term health condition		
Yes	302	34·6
No	571	65·4
Number of GP visits in previous 12 months		
0	273	31·3
1–2	338	38·7
3–10	249	28·5
10 or more	13	1·5
Number of antibiotic prescriptions in last 12 months		
0	626	71·7
1	154	17·6
2	52	6·0
3–5	30	3·4
6+	11	1·3

### Characterisation and incidence of infection syndromes

A total of 40,590 symptoms were reported, with at least one symptom reported by 689/873 (79%) participants. The number and percentage of syndromes in which each symptom was reported at any time are shown in Table S2.

By combining symptoms of the same category into infection syndromes, we identified a total of 1422 infection syndromes (814 respiratory, 222 gastrointestinal, 123 mouth/dental, 111 skin/soft tissue, 87 urinary tract, and 65 eye). Crude and age- and sex-adjusted incidence rates are shown in Table 2 (age and sex-specific rates in Table S3). The age and sex-adjusted incidence rates (and 95% CIs) per person-year were: respiratory 2·03 (1·83–2·26); gastrointestinal 0.68 (0·55–0·83); mouth/dental 0·29 (0·22–0·38); skin/soft tissue 0·23 (0·18–0·30); urinary tract 0·17 (0·11–0·25), and eye 0·14 (0·10–0·20). Respiratory tract infection syndromes showed a winter peak in incidence but there were no clear seasonal variations for other infection syndromes (Figure S3).

**Table 2:** Infection syndrome incidence, GP (or dentist) consultation frequencies and antibiotic use

Infection syndrome	Incident infection syndromes (Total 1422)	Number reported (% of syndromes)		Incidence per person-year (95% confidence interval)	
		GP (or dentist^**a**^) consultations	Any antibiotic^b^	Crude	Age and sex-adjusted
Respiratory	814	75 (9·2)	52 (6·4)	1·98 (1·82–2·14)	2·03 (1·83–2·26)
Gastrointestinal	222	16 (7·2)	2 (0·9)	0·54 (0·46–0·62)	0·68 (0·55–0·83)
Mouth/Dental	123	35 (28·5)	12 (9·8)	0·30 (0·25–0·36)	0·29 (0·22–0·38)
Skin/Soft tissue	111	30 (27·0)	25 (22·5)	0·27 (0·22–0·33)	0·23 (0·18–0·30)
Urinary tract	87	31 (35·6)	36 (41·4)	0·21 (0·16–0·28)	0·17 (0·11–0·25)
Eye	65	7 (10·8)	9 (13·8)	0·16 (0·12–0·21)	0·14 (0·10–0·20)

^a^Mouth/Dental infections only

^b^Any antibiotic prescribed or used for symptoms during infection syndrome (including those from sources other than the GP, see Fig. 2)

### Healthcare-seeking behaviours and antibiotic use

**Fig. 1:**
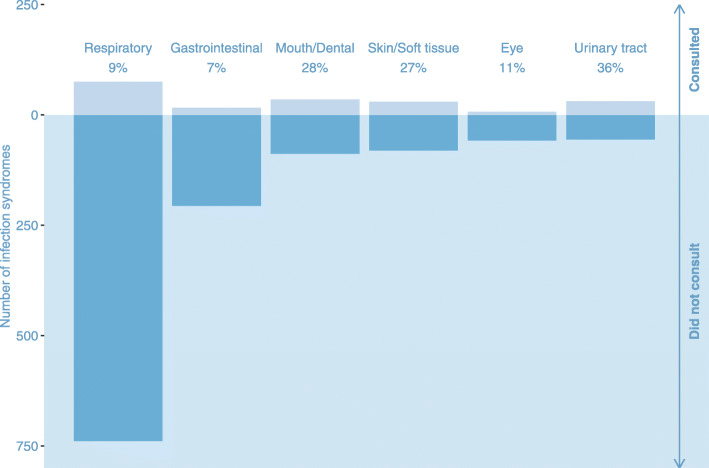
Clinical ‘icebergs’: Number and proportion of infection syndromes leading to GP (or dentist) consultation

The proportions of infection syndromes for which healthcare advice was sought from different sources are shown in Table S4. Overall, GP consultation (in person) was reported most frequently (10% of all infection syndromes, 137/1422), followed by visiting the pharmacy (7%, 101/1422), finding information on the internet (6%, 83/1422), and going to hospital (4%, 59/1422). Few people reported using NHS 111, walk-in centres or an online doctor service. The numbers and proportions of infection syndromes that led to any GP (in person or telephone) or dentist consultation are shown in Fig. 1 and Table 2.

Antibiotic use for incident infection syndromes was reported 168 times by 116/873 (13%) individuals. Overall, 10% (136/1422) infection syndromes led to use of at least one antibiotic. The median number of days between the first symptom and first prescription of an antibiotic was three (interquartile range (IQR) 1–7). Antibiotic use was highest for urinary tract syndromes, for which 41% (36/87) syndromes resulted in at least one antibiotic used (Table 2).

**Fig. 2:**
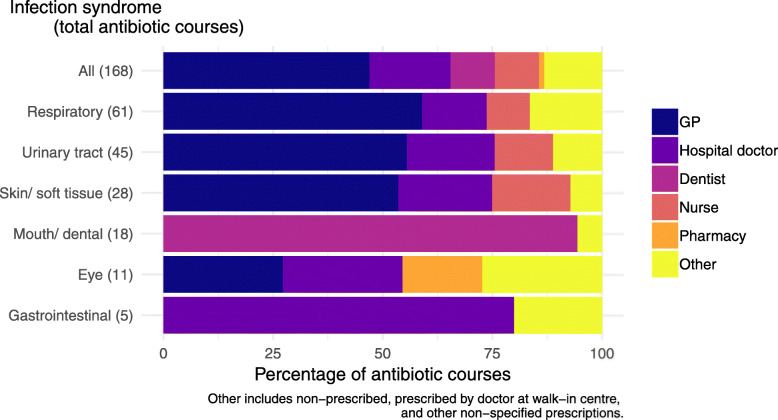
Sources of antibiotics by infection syndrome

Antibiotics were most often obtained from GP prescriptions (79/168, 47%), followed by hospital doctors (31/168, 18%) and dentists (17/168, 10%) (Fig. 2). There were two reports of using antibiotics that were not prescribed for current symptoms (for example those left over from another time). Amoxicillin was the most frequently reported drug (Table S5). The majority of antibiotic prescriptions were for a course of up to 1 week (136/152, 89%, where the course length was reported). Most antibiotics were also reported as taken for up to 1 week (112/168, 67%). Four participants reported taking an antibiotic for 4 weeks or longer; the longest duration reported was 21 weeks (for respiratory tract symptoms). Delayed prescriptions were reported five times and on four occasions people reported not finishing their course of antibiotics; reasons given were side effects, being told to stop by their doctor, and that they were feeling better.

### Symptom duration and impact on health-related quality of life

**Fig. 3:**
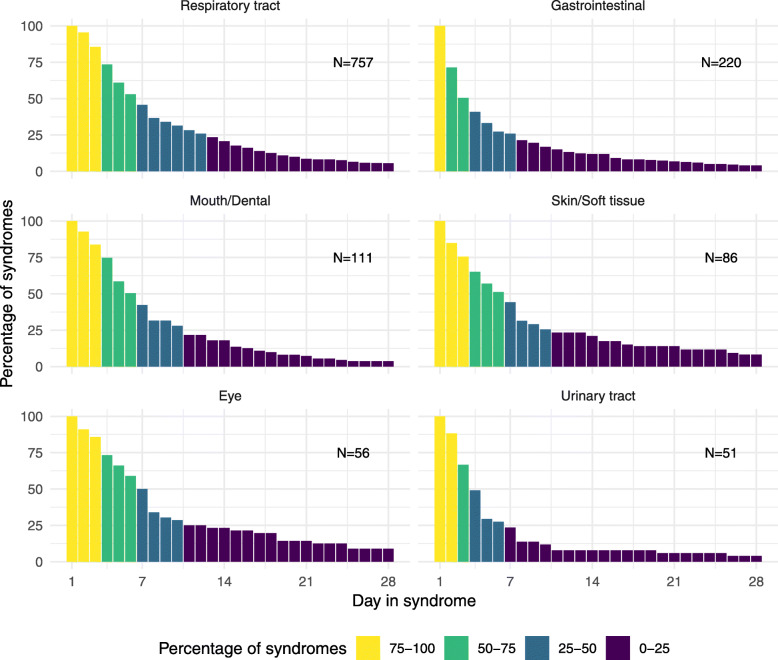
Proportion of infection syndromes ongoing by day

There were 1255 infection syndromes which did not lead to antibiotic use. At least a quarter of infection syndromes of all categories except urinary tract and gastrointestinal lasted into a second week (Fig. 3). The proportion of respiratory infection syndromes that lasted more than 1 week was 37%, whereas 51% of gastrointestinal infection syndromes had resolved before the third day. The number of consecutive days of reporting of specific symptoms are shown in Table S6 and Figure S4.

The estimated mean duration of syndromes, worst day and estimated QALD loss are summarised in Tables 3 and 4. The worst mean recorded day by EQ-5D-3L index was between the second and third day for all syndromes except eye (6 days). All types of syndromes had an estimated mean of less than one total QALD loss per syndrome, except mouth/dental (1·3 days). There was insufficient power to detect a QALD impact for eye infection syndromes. Mean overall and worst EQ-5D-3L index scores are summarised in Table S9.

**Table 3:** Duration of syndromes not treated with antibiotics

Infection syndrome	Number of syndromes^**a**^ (Total 1286)	Crude		Adjusted for right-censoring^c^	
		Median (IQR) days duration	Mean (sd) days duration	Median (IQR) days duration	Mean (95% CI) days duration
Respiratory tract^b^	762	6 (3–11)	9·8 (13·1)	6·8 (3·8–12·1)	11·2 (9·5–13·2)
Gastrointestinal	220	3 (1–7)	8·1 (21·5)	3·1 (1·5–6·4)	7·1 (5·3–9·4)
Mouth/Dental	111	6 (3–10)	9·6 (17·9)	5·8 (3·3–10·3)	9·5 (7·4–12·1)
Skin/Soft tissue	86	6 (3–9)	11·7 (19·9)	5·6 (2·6–12·3)	15·9 (8·8–28·7)
Eye	56	6 (3–10)	14·4 (29·2)	7·5 (3·7–15·1)	16·7 (9·3–29·9)
Urinary tract	51	3 (2–6)	5·9 (8·3)	3·6 (2·1–6·2)	5·6 (4·0–8·0)

*CI* Confidence interval, *IQR* Interquartile range, *sd* Standard deviation

^**a**^Number of syndromes used to estimate duration (excludes syndromes treated with antibiotics)

^b^Age standardised

^c^Based on results from log-logistic models (see Additional File 1)

**Table 4:** Impacts on adult health-related quality of life

Infection syndrome	Number of infection syndromes^**a**^ (Total 1131)	Worst day in syndrome by EQ. 5D index score, mean (s.d)	QALD loss per day, mean (s.d.)	QALD loss per syndrome^**c**^	
				Mean (95% CI)	Median (IQR)
Respiratory tract^b^	663	3·2 (6·9)	0·06 (0·28)	0·7 (0·6–0·8)	0·4 (0·2–0·7)
Gastrointestinal	188	3·0 (9·7)	0·06 (0·34)	0·4 (0·3–0·5)	0·2 (0·1–0·4)
Mouth/Dental	102	3·5 (6·4)	0·14 (0·27)	1·3 (1·1–1·7)	0·8 (0·5–1·5)
Skin/Soft tissue	77	3·3 (6·1)	0·02 (0·29)	0·3 (0·2–0·6)	0·1 (0·1–0·3)
Eye	52	6·0 (16·0)	-0·01 (0·28)	-0·1 (0--0·2)	-0·1 (0--0·1)
Urinary tract	49	2·7 (5·9)	0·09 (0·09)	0·5 (0·3–0·7)	0·3 (0·2–0·5)

*CI* Confidence interval, *IQR* Interquartile range, *QALD* Quality-adjusted life day, *sd* Standard deviation

^**a**^Number of syndromes used to estimate duration (excludes syndromes treated with antibiotics, and those for children under 16)

^b^ Age standardised

^c^Calculated by multiplying the mean QALD loss per day by the mean and median (respectively) duration estimates adjusted for right-censoring (Table 3)

## Discussion

We have measured incidence rates, frequencies of healthcare seeking behaviours and antibiotic use, the average duration of symptoms, and the impacts on health-related quality of life for a range of common acute infection syndromes in the community. Using a novel and efficient method of data collection, we have captured reports of over 40,000 symptoms of respiratory, gastrointestinal, mouth/dental, skin/soft tissue, urinary tract, and eye infections across more than 440 person-years of follow up. Most people did not seek medical advice about their symptoms and one in ten infection syndromes led to antibiotic use. A considerable proportion of people went to hospital for their symptoms (particularly for urinary tract infection syndromes) but the NHS111 service was not used often. Symptoms usually resolved within a week with the worst day of symptoms on average after 3 days.

To our knowledge, this is the first study to measure community-level incidence rates across a range of infection syndromes, and there are no comparable estimates from equivalent large-scales studies for most of the types of syndromes covered. The incidence of respiratory tract infection has been estimated in various settings [10, 23–25] including two large UK cohorts (Flu Survey [23] and Flu Watch [10]). Although these two studies used slightly different case definitions to Bug Watch, and covered winter months rather than the whole year, the estimates were comparable: 3·3 episodes of influenza-like illness per person year (age-standardised) in Flu Survey [23]; 1.7 episodes of any respiratory illness (crude) in Flu Watch [10], and 2·03 syndromes per person year (age and sex-adjusted) in Bug Watch. The IID2 study of infectious intestinal disease in the UK estimated a rate of 0·52 episodes per person year (definite and probable cases) [11], similar to our estimate of 0·68 gastrointestinal syndromes per person year.

We found substantial clinical “icebergs” for all infection types in Bug Watch, with most syndromes not leading to medical consultation. This pattern has been observed previously for respiratory tract [10, 24, 26–28] and gastrointestinal [11, 29] infections, but contrasts with evidence from a cross-sectional study of urinary tract infections which found that 65% adult females in England contacted a GP about their most recent episode [30]. It is likely that this difference is due to omission of less severe cases from the previous study, which involved a one-off interview and relied on recall rather than prospective symptom reporting. Pharmacies and the internet were the other most frequently used sources of information, but the NHS 111 service was not used often. These findings suggest that interventions encouraging safe self-management, for example provision of better information online and in pharmacies, may be effective across all infection categories. Further work could also explore how to promote NHS 111, or to develop online services with access to advice from nurses or pharmacists, which could reduce use of GPs or Accident and Emergency services for these syndromes.

Although most antibiotics reported in Bug Watch were prescribed by GPs, a substantial proportion were from hospital doctors and dentists (for mouth/dental infections). This suggests that some patients may be bypassing primary care services and highlights the need for continued attention to antibiotic stewardship for acute infections across healthcare settings. Despite evidence that delayed prescriptions for respiratory tract infections can reduce use of antibiotics with no difference in symptoms or patient satisfaction [31], they were used infrequently. Half of the antibiotics reported were prescribed within 3 days of the onset of symptoms. There may therefore be scope for wider use of “safety netting” stewardship interventions that give GPs flexibility not to have to prescribe an antibiotic immediately without compromising patient safety.

We have estimated average symptom duration and impacts on health-related quality of life for a range of infection syndromes in the community. For respiratory tract infections, our estimates were comparable to those from Flu Watch (crude mean 9·8 days duration in Bug Watch; 9·0 days for influenza-like-illness and 6·9 days for other acute respiratory tract infections in Flu Watch; mean 0·7 QALD lost per syndrome in Bug Watch; 0·93 for influenza-like illness and 0·26 for other acute respiratory tract infections in Flu Watch) [14]. Other previous estimates of duration have tended to be based on patients who initially presented at primary care, and are therefore likely to have excluded less severe cases. This includes estimates of lower respiratory tract infection (average duration 3 weeks [32]) and urinary tract infection (mean 5 days of symptoms after GP consultation for infections not treated with antibiotics [33]). To our knowledge there are no other community-based estimates of QALD loss for other infection types. We have also provided data on the frequency and duration of individual symptoms of infection. These estimates will be useful in informing messages about symptom expectation for antibiotic stewardship campaigns and for modelling cost effectiveness of potential interventions. In the context of emerging infections such as COVID-19, knowledge of the background frequency of symptoms characteristic of the new infection (Table S10) could be used to inform public health advice regarding testing and self-isolation. For example, data from Bug Watch have been used to estimate the baseline demand for COVID-19 tests due to background cases of cough and fever [34]. All data from the Bug Watch study are available so that different combinations of symptoms can be assessed in future studies [35].

A limitation of this study was that a relatively low proportion of people from the HSE sample responded to the invitation to participate, and some did not consent to be re-contacted about future research. The results may therefore not be representative, with participants more likely to be older, female, and living in less deprived areas than the general population. To partially account for this, we adjusted incidence rate estimates to the age and sex structure of England and reported healthcare-seeking behaviours separately for adults and children and by sex. Whilst there are likely to be differences in healthcare-seeking behaviours for groups not well represented in this study, our data provides community-level information that is not easily obtained from other study designs. Further work is needed to understand how to engage a more diverse population in future similar studies. All surveys for children were completed by their parents/guardians, and healthcare-seeking behaviours are likely to have different drivers for this group, which we plan to explore through future qualitative interviews [36].

It is also possible that people who were motivated to sign up to participate in Bug Watch tended to have more infections or have a particular interest in antibiotic stewardship. Comparisons with the Wellcome Trust Monitor survey suggested that the Bug Watch cohort had a greater awareness of antibiotic resistance but that it was not reflected in large differences in behaviour in terms of antibiotic use. Once registered, the retention of the cohort was good, with 72% of people who signed up completing at least three quarters of their weekly surveys. This suggests that the online survey methodology was acceptable and not too burdensome, so should be considered for future similar studies. Our measures to maintain engagement in the study included provision of regular newsletters with study updates and small tokens of appreciation (£5 vouchers) for those who completed the majority of surveys.

We combined self-reported symptoms of infection on different days into infection syndromes. This relied on assumptions about the plausible length of time between symptoms that could have been caused by the same infection, and could have led to over- or under- ascertainment of incidence rates. Although we asked participants to only report symptoms that they suspected to be caused by an acute infection, there was no microbiological confirmation and it is likely that some symptoms (for example symptoms due to chronic conditions or allergies like hay fever) were mistakenly reported, leading to overestimation of rates. Similarly, the self-reports of healthcare-seeking behaviours were not corroborated with health records, we do not have information on overnight hospitalisation or death, and reports rely on participants correctly interpreting the survey questions. For example, if a participant initially phoned the NHS 111 service but were subsequently transferred to a telephone GP, we cannot be sure if they would have included both behaviours when completing the survey.

## Conclusions

Most people are able to manage symptoms of common infection without medical consultation. The information on consultation and prescribing frequencies and symptom duration will be useful in the design of national and international antibiotic stewardship programs, public health campaigns, and to support GPs in communicating with patients about infections. Interventions encouraging safe self-management across a range of acute infection syndromes could decrease pressure on primary healthcare services and support targets for reducing antibiotic prescribing. Our health-related quality of life estimates could be used to in health economic models of the impacts of interventions. The novel data on symptom duration and frequency that we have captured is also valuable for assessing characteristics of emerging infections such as COVID-19 [34].

## Supplementary Information

**Additional file 1 Table S1.** Baseline characteristics and population comparisons. **Table S2.** Symptom reporting by infection syndrome. **Table S3.** Age and sex-specific rates of infection syndromes. **Table S4.** Healthcare-seeking behaviours by type of infection and sex for adults and children. **Table S5.** Reported types of antibiotics used by infection syndrome. **Table S6.** Consecutive days of symptom reporting. **Table S7.** Symptom duration model fitting by model and infection syndrome. **Table S8.** Age-specific estimates of symptom duration for respiratory infection syndromes. **Table S9.** Health-related quality of life at baseline. **Table S10.** Health-related quality of life impacts by infection syndrome. **Table S11.** Background frequency of symptoms related to novel coronavirus (COVID-19) in Bug Watch cohort by day and month. **Figure S1.** Identification of incident infection syndromes. **Figure S2.** Number of complete and incomplete weekly surveys. **Figure S3.** Inclusion and exclusion of study participants. **Figure S4.** Infection syndrome incidence by month. **Figure S5.** Symptom reporting by infection syndrome.

## Data Availability

All data and code for analyses are available on the UK Data Service (10.5255/UKDA-SN-8734-1).

## Abbreviations

AMR: Antimicrobial resistance
CI: Confidence interval
EQ-5D-3L: Euroqol - 5 Dimensions - 3 Levels
GP: General practitioner
HSE: Health Survey for England
NHS: National Health Service
QALD: Quality-adjusted life day
