# Supplementary material for: Incidence, healthcare-seeking behaviours, antibiotic use and natural history of common infection syndromes in England: results from the Bug Watch community cohort study
Source: BMC Infect Dis. 2021 Jan 22;21:105. doi: 10.1186/s12879-021-05811-7 (PMC7820521; doi:10.1186/s12879-021-05811-7)
Supplement: Supplementary file 1 — Additional file 1 Table S1. Baseline characteristics and population comparisons. Table S2. Symptom reporting by infection syndrome. Table S3. Age and sex-specific rates of infection syndromes. Table S4. Healthcare-seeking behaviours by type of infection and sex for adults and children. Table S5. Reported types of antibiotics used by infection syndrome. Table S6. Consecutive days of symptom reporting. Table S7. Symptom duration model fitting by model and infection syndrome. Table S8. Age-specific estimates of symptom duration for respiratory infection syndromes. Table S9. Health-related quality of life at baseline. Table S10. Health-related quality of life impacts by infection syndrome. Table S11. Background frequency of symptoms related to novel coronavirus (COVID-19) in Bug Watch cohort by day and month. Figure S1. Identification of incident infection syndromes. Figure S2. Number of complete and incomplete weekly surveys. Figure S3. Inclusion and exclusion of study participants. Figure S4. Infection syndrome incidence by month. Figure S5. Symptom reporting by infection syndrome. [file 12879_2021_5811_MOESM1_ESM.docx]

Supplementary material: Incidence, healthcare-seeking behaviours, antibiotic use, and natural history of common infection syndromes in England: Results from the Bug Watch community cohort study

##

## Table S1: Baseline characteristics and population comparisons

|  | **Included in cohort** | | **Excluded from cohort** | |  |
| --- | --- | --- | --- | --- | --- |
| **Variable, source of population comparison** | **N /denominator for given question***  **(Total 873)** | **%** | **N /denominator for given question**  **(Total 369)** | **%** | **Population %** |
| **Demographics** |  |  |  |  |  |
| Sex, Office for National Statistics mid-2017 population estimates |  |  |  |  |  |
| Male | 343/873 | 39·3 | 160/369 | 43·7 | 49·4 |
| Female | 528/873 | 60·5 | 206/369 | 56·3 | 50·6 |
| Unspecified or prefer not to say | 2/873 | 0·2 |  |  |  |
| Age group (years), Office for National Statistics mid-2017 population estimates |  |  |  |  |  |
| 5 or less | 26/873 | 3·0 | 18/369 | 4·9 | 7·3 |
| 6-16 | 65/873 | 7·5 | 51/369 | 13·9 | 12·8 |
| 17-35 | 57/873 | 6·5 | 67/369 | 18·3 | 24·7 |
| 36-55 | 170/873 | 19·5 | 92/369 | 25·1 | 26·6 |
| 56-70 | 377/873 | 43·2 | 101/369 | 27·6 | 16·8 |
| 71+ | 174/873 | 19·9 | 35/369 | 9·6 | 11·7 |
| Region, Office for National Statistics mid-2017 population estimates |  |  |  |  |  |
| East Midlands | 81/873 | 9·3 | 32/369 | 8·7 | 8·6 |
| East of England | 130/873 | 14·9 | 41/369 | 11·2 | 11·1 |
| London | 73/873 | 8·4 | 41/369 | 11·2 | 15·9 |
| North East | 60/873 | 6·9 | 28/369 | 7·7 | 4·8 |
| North West | 92/873 | 10·5 | 49/369 | 13·4 | 13·1 |
| South East | 172/873 | 19·7 | 68/369 | 18·6 | 16·3 |
| South West | 100/873 | 11·5 | 26/369 | 7·1 | 10·0 |
| West Midlands | 93/873 | 10·7 | 40/369 | 10·9 | 10·5 |
| Yorkshire and the Humber | 69/873 | 7·9 | 37/369 | 10·1 | 9·8 |
| Index of multiple deprivation quintile, Office for National Statistics mid-2017 population estimates |  |  |  |  |  |
| 1 (20% most deprived) | 49/873 | 5·6 | 73/369 | 20·0 | 20·0 |
| 2 | 155/873 | 17·8 | 56/369 | 15·3 | 20·0 |
| 3 | 206/873 | 23·6 | 68/369 | 18·6 | 20·0 |
| 4 | 218/873 | 25·0 | 94/369 | 25·7 | 20·0 |
| 5 (20% least deprived) | 242/873 | 27·7 | 71/369 | 19·4 | 20·0 |
| Ethnic group, Office for National Statistics 2011 population estimates |  |  |  |  |  |
| White | 839/873 | 96·1 | 313/369 | 85·5 | 85·4 |
| Black | 4/873 | 0·5 | 8/369 | 2·2 | 3·5 |
| Asian | 12/873 | 1·4 | 28/369 | 7·7 | 7·7 |
| Mixed/ multiple ethnic background | 10/873 | 1·1 | 8/369 | 2·2 | 2·3 |
| Any other ethnic group | 7/873 | 0·8 | 4/369 | 1·1 | 1·0 |
| Born in the UK |  |  |  |  |  |
| Yes | 821/873 | 94·0 | 327/369 | 89·3 |  |
| No | 52/873 | 6·0 | 39/369 | 10·7 |  |
| Place of birth (other than UK) |  |  |  |  |  |
| Africa | 11/873 | 1·3 | 10/369 | 2·7 |  |
| Americas | 7/873 | 0·8 | 5/369 | 1·4 |  |
| Asia | 10/873 | 1·1 | 15/369 | 4·1 |  |
| Europe | 20/873 | 2·3 | 8/369 | 2·2 |  |
| Oceania | 4/873 | 0·5 | 1/369 | 0·3 |  |
| Work and school |  |  |  |  |  |
| Economic activity, Health Survey for England 2014 |  |  |  |  |  |
| In employment | 333/873 | 38·1 | 167/369 | 45·6 | 45·5 |
| Retired | 376/873 | 43·1 | 73/369 | 20·0 | 17·7 |
| Other economically inactive | 69/873 | 7·9 | 57/369 | 15·6 | 13·6 |
| Type of employment |  |  |  |  |  |
| Full-time | 214/873 | 24·5 | 131/369 | 35·8 |  |
| Part-time | 120/873 | 13·7 | 39/369 | 10·7 |  |
| Healthcare worker |  |  |  |  |  |
| Yes | 52/873 | 6·0 | 23/369 | 6·3 |  |
| No | 282/873 | 32·3 | 147/369 | 40·2 |  |
| In school |  |  |  |  |  |
| Yes | 16/873 | 1·8 | 11/369 | 3 |  |
| No | 5/873 | 0·6 | 4/369 | 1·1 |  |
| **Medical** |  |  |  |  |  |
| Long-term health condition† |  |  |  |  |  |
| Chronic lung disease | 57/873 | 6·5 | 36/369 | 9·8 |  |
| Chronic heart disease | 29/873 | 3·3 | 12/369 | 3·3 |  |
| Heart problems from birth | 4/873 | 0·5 | 6/369 | 1·6 |  |
| Chronic kidney disease | 7/873 | 0·8 | 3/369 | 0·8 |  |
| Diabetes | 45/873 | 5·2 | 20/369 | 5·5 |  |
| Chronic liver disease | 2/873 | 0·2 | 1/369 | 0·3 |  |
| Regular abdominal pain or bloating | 45/873 | 5·2 | 20/369 | 5·5 |  |
| Chronic skin problems | 22/873 | 2·5 | 15/369 | 4·1 |  |
| Chemotherapy for cancer | 5/873 | 0·6 | 3/369 | 0·8 |  |
| Neurological disorder | 38/873 | 4·4 | 16/369 | 4·4 |  |
| Mental health disorder | 20/873 | 2·3 | 21/369 | 5·7 |  |
| Other long term condition | 174/873 | 19·9 | 60/369 | 16·4 |  |
| Currently pregnant |  |  |  |  |  |
| Yes | 1/873 | 0·1 | 1/369 | 0·3 |  |
| No | 468/873 | 53·6 | 172/369 | 47·0 |  |
| Smoking status |  |  |  |  |  |
| Yes | 32/873 | 3·7 | 35/369 | 9·6 |  |
| No - used to smoke | 294/873 | 33·7 | 110/369 | 30·1 |  |
| No - never smoked | 456/873 | 52·2 | 154/369 | 42·1 |  |
| Number of GP visits in previous 12 months |  |  |  |  |  |
| 0 | 273/873 | 31·3 | 100/369 | 27·3 |  |
| 1-2 | 338/873 | 38·7 | 134/369 | 36·6 |  |
| 3-10 | 249/873 | 28·5 | 125/369 | 34·2 |  |
| 10 or more | 13/873 | 1·5 | 7/369 | 1·9 |  |
| Influenza vaccination in last 12 months |  |  |  |  |  |
| Yes | 472/873 | 54. ·1 | 166/369 | 45·4 |  |
| No | 396/873 | 45·4 | 195/369 | 53·3 |  |
| Not sure | 5/873 | 0·6 | 5/369 | 1·4 |  |
| Up to date with childhood vaccinations |  |  |  |  |  |
| Yes | 88/91 | 96·7 | 60/67 | 89·6 |  |
| No | 3/91 | 3·3 | 4/7 | 6·0 |  |
| **Antibiotics, Wellcome Trust Monitor Survey** |  |  |  |  |  |
| Ever prescribed antibiotics |  |  |  |  |  |
| Yes | 783/873 | 89·7 | 307/366 | 83·9 | 91 |
| No | 43/873 | 4·9 | 36/366 | 9·8 | 6 |
| Think so, but am not sure | 38/873 | 4·4 | 15/366 | 4·1 | 3 |
| Can’t remember | 9/873 | 1·0 | 8/366 | 2·2 | 1 |
| Number of antibiotic prescriptions in last 12 months |  |  |  |  |  |
| 0 | 626/873 | 71·7 | 237/366 | 64·8 |  |
| 1 | 154/873 | 17·6 | 68/366 | 18·6 |  |
| 2 | 52/873 | 6·0 | 30/366 | 8·2 |  |
| 3-5 | 30/873 | 3·4 | 22/366 | 6·0 |  |
| 6+ | 11/873 | 1·3 | 9/366 | 2·5 |  |
| Ever prescribed antibiotics and thought inappropriate |  |  |  |  |  |
| Yes | 59/782 | 7·5 | 36/307 | 11·7 | 22 |
| No | 724/782 | 92·5 | 271/307 | 88·3 | 77 |
| When they last took antibiotics |  |  |  |  |  |
| Can’t remember | 77/782 | 9·8 | 42/307 | 13·7 | 2 |
| Within the last month | 46/782 | 5·9 | 26/307 | 8·5 | 11 |
| Within the last three months | 65/782 | 8·3 | 30/307 | 9·8 | 11 |
| Within the last year | 134/782 | 17·1 | 66/307 | 21·5 | 23 |
| Within the last two years | 134/782 | 17·1 | 48/307 | 15·6 | 16 |
| More than two years ago | 327/782 | 41·8 | 95/307 | 31·0 | 37 |
| Were their last antibiotics prescribed |  |  |  |  |  |
| Yes | 696/706 | 98·6 | 264/265 | 99·6 | 99 |
| No | 3/706 | 0·4 |  |  | 0·4 |
| Can’t remember | 7/706 | 1·0 | 1/265 | 0·4 | 0·1 |
| What they did with their last prescription |  |  |  |  |  |
| Can’t remember | 6/696 | 0·9 |  |  | 1 |
| Took all the antibiotics prescribed, at the right times | 646/696 | 92·8 | 236/264 | 89·4 | 86 |
| Took all the antibiotics prescribed, but not at the right times | 12/696 | 1·7 | 11/264 | 4·2 | 7 |
| Did not take all the antibiotics prescribed | 17/696 | 2·4 | 13/264 | 4·9 | 5 |
| Currently taking antibiotics | 12/696 | 1·7 | 3/264 | 1·1 | 1 |
| Other | 3/696 | 0·4 | 1/264 | 0·4 | 0·8 |
| Why didn't they complete the antibiotic course† |  |  |  |  |  |
| Forgot to take them | 2/17 | 11·8 | 2/13 | 15·4 | 12 |
| Was feeling better | 9/17 | 52·9 | 5/13 | 38·5 | 60 |
| Started to get side-effects | 3/17 | 17·6 | 3/13 | 23·1 | 25 |
| Did not replace doses missed | 1/17 | 5·9 | 1/13 | 7·7 | 1 |
| Wanted to drink alcohol | 1/17 | 5·9 | 1/13 | 7·7 | 1 |
| Wanted to save them in case got ill again | 3/17 | 17·6 | 1/13 | 7·7 | 1 |
| Other | 6/17 | 35·3 | 4/13 | 15·4 | 4 |
| Where they got non-prescribed antibiotics† |  |  |  |  |  |
| Got them abroad | 1/3 | 33·3 |  |  | 42 |
| Other | 2/3 | 66·7 |  |  |  |
| Ever asked for antibiotics to be prescribed |  |  |  |  |  |
| Yes | 100/873 | 11·5 | 51/366 | 13·9 | 21 |
| No | 753/873 | 86·3 | 297/366 | 81·1 | 78 |
| Can’t remember | 20/873 | 2·3 | 18/366 | 4·9 | 1 |
| Were they given a prescription when asked |  |  |  |  |  |
| Yes | 93/100 | 93 | 46/51 | 90·2 | 85 |
| No | 7/100 | 7 | 3/51 | 5·9 | 14 |
| Had to persuade GP to get prescription |  |  | 2/51 | 3·9 |  |
| Yes | 14/93 | 15·1 | 4/46 | 8·7 | 16 |
| No | 79/93 | 85·0 | 39/46 | 84·8 | 84 |
| Tried to persuade GP to prescribe but not prescribed |  |  | 3/46 | 6·5 |  |
| Yes | 2/7 | 28·6 | 2/3 | 66·7 | 25 |
| No | 5/7 | 71·4 | 1/3 | 33·3 | 75 |
| Which conditions can be effectively treated by antibiotics† |  |  |  |  |  |
| Viral | 129/782 | 16·5 | 96/299 | 32·1 | 38 |
| Fungal | 88/782 | 11·3 | 63/299 | 21·1 | 21 |
| Bacterial | 729/782 | 93·2 | 261/299 | 87·3 | 84 |
| Cold | 2/782 | 0·3 | 12/299 | 4·0 | 8 |
| Flu | 30/782 | 3·8 | 29/299 | 9·7 | 15 |
| Allergies | 38/782 | 4·9 | 19/299 | 6·4 | 9 |
| Other/Don’t know | 19/782 | 2·4 | 15/299 | 5·0 | 2 |
| Bacterial only | 556/782 | 71·1 | 148/299 | 49·5 | 41 |
| Level of understanding of the term "antibiotic resistance" |  |  |  |  |  |
| Very good | 281/782 | 35·9 | 89/299 | 29·8 | 24 |
| Good | 269/782 | 34·4 | 88/299 | 29·4 | 31 |
| Some understanding | 182/782 | 23·3 | 74/299 | 24·7 | 25 |
| Have heard the term but little understanding of what it means | 36/782 | 4·6 | 25/299 | 8·4 | 11 |
| Have not heard the term/ Don’t know | 14/782 | 1·8 | 23/299 | 7·7 | 9 |

*based on skip logic from Wellcome Trust Monitor survey.

†checkbox variables, therefore percentages may sum to more than 100.

## Table S2: Symptom reporting by infection syndrome

| **Category (total infection syndromes) (Total 1,422)** | **Specific symptoms** | **N (%) syndromes** | **Non-specific symptoms** | **N (%) syndromes** |
| --- | --- | --- | --- | --- |
| Respiratory tract (814) | Runny nose | 478 (59) | Headache | 222 (27) |
|  | Sore throat | 474 (58) | Fatigue | 206 (25) |
|  | Sneezing | 428 (53) | Muscle aches | 155 (19) |
|  | Blocked nose | 346 (43) | Fever | 119 (15) |
|  | Dry cough | 340 (42) | Chills | 95 (12) |
|  | Coughing up phlegm | 322 (40) | Loss of appetite | 75 (9) |
|  | Sinus pain | 173 (21) | Night sweats | 65 (8) |
|  | Shortness of breath | 111 (14) | Migraine | 15 (2) |
|  | Ear ache | 95 (12) |  |  |
|  | Ear fluid | 9 (1) |  |  |
| Gastrointestinal (222) | Diarrhoea | 139 (63) | Fatigue | 48 (22) |
|  | Stomach pain | 94 (42) | Headache | 44 (20) |
|  | Vomiting | 74 (33) | Muscle aches | 38 (17) |
|  | Nausea | 63 (28) | Loss of appetite | 35 (16) |
|  |  |  | Fever | 30 (14) |
|  |  |  | Chills | 22 (10) |
|  |  |  | Night sweats | 15 (7) |
|  |  |  | Migraine | 11 (5) |
| Mouth/Dental (123) | Mouth ulcer | 62 (50) | Muscle aches | 17 (14) |
|  | Toothache | 48 (39) | Fatigue | 16 (13) |
|  | Gum abscess | 27 (22) | Headache | 16 (13) |
|  |  |  | Loss of appetite | 8 (7) |
|  |  |  | Chills | 6 (5) |
|  |  |  | Fever | 5 (4) |
|  |  |  | Migraine | 5 (4) |
|  |  |  | Night sweats | 3 (2) |
| Skin/Soft tissue (111) | Rash (local) | 49 (44) | Fatigue | 15 (14) |
|  | Itchy (local) | 44 (40) | Muscle aches | 12 (11) |
|  | Infected wound | 37 (33) | Headache | 10 (9) |
|  | Boil | 8 (7) | Loss of appetite | 8 (7) |
|  | Itchy (all over) | 6 (5) | Chills | 8 (7) |
|  | Rash (all over) | 4 (4) | Fever | 8 (7) |
|  | Mastitis | 2 (2) | Night sweats | 8 (7) |
|  | Shingles | 2 (2) | Migraine | 2 (2) |
| Urinary tract (87) | Frequent urination | 57 (66) | Fatigue | 14 (16) |
|  | Painful urination | 46 (53) | Headache | 11 (13) |
|  | Urgent urination | 44 (51) | Loss of appetite | 10 (11) |
|  | Dark urine | 32 (37) | Muscle aches | 9 (10) |
|  | Bladder pain | 30 (34) | Fever | 5 (6) |
|  | Kidney pain | 21 (24) | Chills | 4 (5) |
|  | Blood in urine | 15 (17) | Night sweats | 2 (2) |
| Eye (65) | Conjunctivitis | 37 (57) | Fatigue | 22 (34) |
|  | Red eye | 21 (32) | Headache | 17 (26) |
|  | Stye | 15 (23) | Muscle aches | 15 (23) |
|  |  |  | Fever | 9 (14) |
|  |  |  | Night sweats | 8 (12) |
|  |  |  | Chills | 7 (11) |
|  |  |  | Loss of appetite | 6 (9) |
|  |  |  | Migraine | 2 (3) |

## Table S3: Age and sex-specific rates of infection syndromes

|  |  | **Age and sex-specific incidence per person-year (95% CI)** | | | | | |
| --- | --- | --- | --- | --- | --- | --- | --- |
| **Infection syndrome** | **Age group (years)** | **5 or less** | **6-16** | **17-35** | **36-55** | **56-70** | **71+** |
| Respiratory | Female | 3·33 (2-5·19) | 1·65 (1·12-2·34) | 3·15 (2·37-4·11) | 2·81 (2·38-3·28) | 2·06 (1·80-2·35) | 1·35 (1·01-1·76) |
|  | Male | 3·31 (2·07-5·01) | 2·28 (1·52-3·3) | 0·92 (0·42-1·74) | 1·32 (0·90-1·87) | 1·79 (1·48-2·14) | 1·34 (1·01-1·74) |
| Gastrointestinal | Female | 1·38 (0·6-2·73) | 0·37 (0·15-0·77) | 1·1 (0·66-1·72) | 0·78 (0·57-1·05) | 0·50 (0·38-0·65) | 0·30 (0·16-0·53) |
|  | Male | 1·95 (1·04-3·33) | 0·33 (0·09-0·84) | 0·91 (0·42-1·72) | 0·33 (0·14-0·64) | 0·41 (0·27-0·59) | 0·33 (0·18-0·55) |
| Skin/Soft tissue | Female | 0·52 (0·11-1·51) | 0·21 (0·06-0·54) | 0·23 (0·06-0·59) | 0·32 (0·19-0·5) | 0·34 (0·24-0·46) | 0·20 (0·09-0·40) |
|  | Male | 0·45 (0·09-1·31) | 0 (0·00-0·30) | 0·20 (0·02-0·73) | 0·04 (0-0·23) | 0·21 (0·12-0·36) | 0·38 (0·21-0·61) |
| Mouth/Dental | Female | 0·52 (0·11-1·51) | 0·16 (0·03-0·47) | 0·64 (0·32-1·14) | 0·45 (0·29-0·66) | 0·30 (0·21-0·42) | 0·20 (0·09-0·40) |
|  | Male | 0·15 (0·00-0·84) | 0·16 (0·02-0·59) | 0 (0·00-0·37) | 0·24 (0·09-0·53) | 0·29 (0·17-0·45) | 0·26 (0·13-0·46) |
| Urinary tract | Female | 0·17 (0-0·96) | 0 (0·00-0·20) | 0·40 (0·16-0·83) | 0·23 (0·12-0·4) | 0·3 (0·21-0·42) | 0·28 (0·14-0·5) |
|  | Male | 0 (0·00-0·55) | 0·08 (0·00-0·45) | 0 (0·00-0·37) | 0·08 (0·01-0·29) | 0·14 (0·06-0·26) | 0·19 (0·08-0·37) |
| Eye | Female | 0·35 (0·04-1·25) | 0 (0·00-0·2) | 0·29 (0·09-0·67) | 0·14 (0·06-0·28) | 0·18 (0·11-0·28) | 0·18 (0·07-0·37) |
|  | Male | 0·3 (0·04-1·08) | 0 (0·00-0·3) | 0 (0·00-0·37) | 0·12 (0·02-0·36) | 0·2 (0·1-0·33) | 0·09 (0·03-0·24) |

###

## Table S4: Healthcare-seeking behaviours by type of infection and sex for adults and children

|  |  | **Number (%) using source** | | | | |
| --- | --- | --- | --- | --- | --- | --- |
|  |  | **Adult** | | **Child (under 16 years)** | | **All participants*** |
|  |  | **Male** | **Female** | **Male** | **Female** |  |
| **Total number of individuals** | | 303 | 474 | 39 | 51 | 873 |
| **Infection syndrome**  **(Total 1,422)** | **Source** |  |  |  |  |  |
| Respiratory | *Incident episodes* | *214* | *495* | *50* | *50* | *814* |
|  | GP (in person) | 11 (5) | 46 (9) | 5 (10) | 4 (8) | 66 (8) |
|  | Pharmacy | 16 (7) | 31 (6) | 1 (2) | 1 (2) | 51 (6) |
|  | Internet information | 6 (3) | 28 (6) | 1 (2) | 1 (2) | 36 (4) |
|  | Hospital | 7 (3) | 6 (1) | 0 | 0 | 13 (2) |
|  | GP (telephone) | 4 (2) | 10 (2) | 1 (2) | 1 (2) | 16 (2) |
|  | Nurse | 2 (1) | 11 (2) | 0 | 0 | 13 (2) |
|  | NHS 111 | 1 (0) | 4 (1) | 0 | 0 | 5 (1) |
|  | Walk-in centre | 0 | 2 (0) | 1 (2) | 1 (2) | 4 (1) |
|  | Internet doctor | 0 | 2 (0) | 0 | 0 | 2 (0) |
| Gastrointestinal | *Incident episodes* | *58* | *132* | *17* | *15* | *222* |
|  | GP (in person) | 3 (5) | 9 (7) | 0 | 0 | 12 (5) |
|  | Pharmacy | 6 (10) | 5 (4) | 0 | 0 | 11 (5) |
|  | Internet information | 1 (2) | 11 (8) | 1 (6) | 2 (13) | 15 (7) |
|  | Hospital | 6 (10) | 6 (5) | 0 | 0 | 12 (5) |
|  | GP (telephone) | 1 (2) | 3 (2) | 1 (6) | 0 | 5 (2) |
|  | NHS 111 | 1 (2) | 3 (2) | 0 | 0 | 2 (1) |
|  | Walk-in centre | 1 (2) | 2 (2) | 1 (6) | 0 | 4 (2) |
|  | Nurse | 0 | 2 (2) | 0 | 0 | 4 (2) |
| Mouth/ dental | *Incident episodes* | *36* | *78* | *0* | *6* | *123* |
|  | GP (in person) | 1 (3) | 1 (1) | 0 | 1 (17) | 3 (2) |
|  | Pharmacy | 4 (11) | 8 (10) | 0 | 1 (17) | 13 (11) |
|  | Internet information | 4 (11) | 3 (4) | 0 | 0 | 7 (6) |
|  | Dentist | 15 (42) | 16 (21) | 0 | 0 | 31 (25) |
|  | Walk-in centre | 1 (3) | 1 (1) | 0 | 0 | 2 (2) |
|  | Hospital | 0 | 2 (3) | 0 | 0 | 2 (2) |
|  | GP (telephone) | 0 | 1 (1) | 0 | 0 | 1 (1) |
|  | Nurse | 0 | 3 (4) | 0 | 0 | 3 (2) |
|  | NHS 111 | 0 | 2 (3) | 0 | 0 | 2 (2) |
| Skin/ soft tissue | *Incident episodes* | *33* | *68* | *3* | *7* | *111* |
|  | GP (in person) | 7 (21) | 20 (29) | 0 | 3 (43) | 30 (27) |
|  | Pharmacy | 3 (9) | 4 (6) | 0 | 0 | 7 (6) |
|  | Internet information | 2 (6) | 13 (19) | 0 | 1 (14) | 16 (14) |
|  | Hospital | 4 (12) | 8 (12) | 0 | 1 (14) | 13 (12) |
|  | GP (telephone) | 1 (3) | 1 (1) | 0 | 0 | 2 (2) |
|  | Nurse | 3 (9) | 5 (7) | 0 | 2 (29) | 10 (9) |
|  | Walk-in centre | 0 | 2 (3) | 1 (33) | 1 (14) | 3 (3) |
|  | NHS 111 | 0 | 2 (3) | 0 | 1 (14) | 4 (4) |
|  | Internet doctor | 0 | 1 (1) | 0 | 0 | 1 (1) |
| Urinary tract | *Incident episodes* | *19* | *65* | *0* | *1* | *87* |
|  | GP (in person) | 2 (11) | 15 (23) | 0 | 1 (100) | 19 (22) |
|  | Internet information | 2 (11) | 6 (9) | 0 | 0 | 8 (9) |
|  | Hospital | 10 (53) | 5 (8) | 0 | 0 | 15 (17) |
|  | GP (telephone) | 3 (16) | 12 (18) | 0 | 0 | 15 (17) |
|  | Nurse | 1 (5) | 7 (11) | 0 | 0 | 8 (9) |
|  | NHS 111 | 1 (5) | 2 (3) | 0 | 0 | 3 (3) |
|  | Pharmacy | 0 | 3 (5) | 0 | 1 (100) | 4 (5) |
|  | Walk-in centre | 0 | 2 (3) | 0 | 0 | 2 (2) |
| Eye | *Incident episodes* | *20* | *41* | *2* | *0* | *65* |
|  | GP (in person) | 1 (5) | 6 (15) | 0 | 0 | 7 (11) |
|  | Pharmacy | 8 (40) | 6 (15) | 1 (50) | 0 | 15 (23) |
|  | Hospital | 2 (10) | 2 (5) | 0 | 0 | 4 (6) |
|  | Internet information | 0 | 1 (2) | 0 | 0 | 1 (2) |
|  | Nurse | 0 | 1 (2) | 0 | 0 | 1 (2) |
|  | NHS 111 | 0 | 2 (5) | 0 | 0 | 2 (3) |

*where totals are larger than column sums this is due to inclusion of syndromes of participants where sex was not reported.

## Table S5: Reported types of antibiotics used by infection syndrome

| **Infection syndrome** | **Antibiotic** | **N reports** |
| --- | --- | --- |
| Respiratory | Amoxicillin | 27 |
|  | Doxycycline | 10 |
|  | Clarithromycin | 6 |
|  | Ciprofloxacin | 3 |
|  | Co-amoxiclav | 3 |
|  | Penicillin | 3 |
|  | Phenoxymethylpenicillin | 2 |
|  | Azithromycin | 1 |
|  | Cefuroxime | 1 |
|  | Chloramphenicol | 1 |
|  | Flucloxacillin | 1 |
|  | Prednisolone | 1 |
| Skin/Soft tissue | Flucloxacillin | 12 |
|  | Fusidic acid | 4 |
|  | Clarithromycin | 2 |
|  | Amoxicillin | 1 |
|  | Diphenhydramine | 1 |
|  | Erythromycin | 1 |
|  | Lymecycline | 1 |
|  | Metronidazole | 1 |
|  | Miconazole | 1 |
|  | Neomycin | 1 |
|  | Teicoplanin | 1 |
| Eye | Chloramphenicol | 4 |
|  | Cefuroxime | 1 |
|  | Doxycycline | 1 |
|  | Erythromycin | 1 |
|  | Flucloxacillin | 1 |
|  | Gentamicin | 1 |
|  | Ofloxacin | 1 |
| Urinary tract | Nitrofurantoin | 23 |
|  | Trimethoprim | 7 |
|  | Cefalexin | 2 |
|  | Ciprofloxacin | 1 |
|  | Cefuroxime | 1 |
|  | Metronidazole | 1 |
| Gastrointestinal | Ciprofloxacin | 1 |
|  | Gentamicin | 1 |
|  | Metronidazole | 1 |
| Mouth/Dental | Amoxicillin | 11 |
|  | Metronidazole | 2 |
|  | Erythromycin | 1 |
|  | Penicillin | 1 |

## Table S6: Consecutive days of symptom reporting

| **Infection syndrome (total syndromes)** | **Specific symptoms** | **Number of consecutive reporting episodes** | **Median number of consecutive days reported (inter-quartile range; 90^th^ percentile)** | **Non-specific symptoms** | **Number of consecutive reporting episodes** | **Median number of consecutive days reported (inter-quartile range; 90^th^ percentile)** |
| --- | --- | --- | --- | --- | --- | --- |
| Respiratory tract  (814) | Runny nose | 546 | 4 (3-7; 10) | Headache | 305 | 2 (1-5; 8) |
|  | Sore throat | 522 | 3 (2-6; 9) | Fatigue | 220 | 4 (3-7; 9) |
|  | Sneezing | 492 | 4 (2-6; 7) | Muscle aches | 187 | 3 (2-7; 9) |
|  | Blocked nose | 390 | 4 (2-7; 10) | Fever | 117 | 4 (2-6; 7) |
|  | Dry cough | 381 | 4 (2-7; 11) | Chills | 99 | 4 (2-5; 7) |
|  | Coughing up phlegm | 359 | 5 (3-7; 13) | Loss of appetite | 80 | 4.5 (3-7; 13) |
|  | Sinus pain | 191 | 4 (2-7; 10) | Night sweats | 70 | 4 (2-7; 12) |
|  | Short of breath | 115 | 4 (2-7; 14) | Migraine | 28 | 2 (1-4; 6) |
|  | Ear ache | 97 | 3 (2-6; 7) |  |  |  |
|  | Ear fluid | 6 | 3 (2-6; 11) |  |  |  |
| Gastrointestinal  (222) | Diarrhoea | 170 | 2 (1-2; 4) | Headache | 101 | 2 (1-4; 8) |
|  | Stomach pain | 138 | 2 (1-3; 7) | Muscle aches | 65 | 3 (2-7; 20) |
|  | Nausea | 76 | 2 (1-3; 6) | Fatigue | 64 | 4 (2-7; 22) |
|  | Vomiting | 75 | 1 (1-2; 2) | Loss of appetite | 52 | 4 (2-7; 12) |
|  |  |  |  | Fever | 32 | 3 (2-5; 7) |
|  |  |  |  | Chills | 27 | 3 (2-4; 7) |
|  |  |  |  | Migraine | 22 | 2 (1-3; 4) |
|  |  |  |  | Night sweats | 19 | 5 (1-8; 29) |
| Urinary tract  (87) | Frequent urination | 34 | 2 (2-4; 4) | Headache | 14 | 1 (1-2; 3) |
|  | Painful urination | 30 | 2 (2-3; 5) | Fatigue | 9 | 2 (2-3; 4) |
|  | Urgent urination | 28 | 2 (2-3; 4) | Muscle aches | 7 | 2 (1-3; 5) |
|  | Dark urine | 20 | 2 (1-3) | Loss of appetite | 6 | 2 (2-4; 5) |
|  | Bladder pain | 17 | 2 (2-3; 4) | Chills | 3 | 1 (1-2; 3) |
|  | Kidney pain | 12 | 4 (1-4; 7) | Fever | 3 | 4 (3-5; 6) |
|  | Blood in urine | 5 | 2 (1-3; 6) | Night sweats | 1 | 3 (3-3; 3) |
| Skin/Soft tissue  (111) | Itchy (local) | 48 | 3 (1-5; 8) | Muscle aches | 17 | 4 (2-7; 24) |
|  | Rash (local) | 44 | 4 (1-6; 10) | Fatigue | 16 | 5 (2-16; 31) |
|  | Infected wound | 30 | 4 (2-7; 13) | Headache | 14 | 2 (2-3; 3) |
|  | Itchy (all over) | 22 | 1 (1-1; 2) | Loss of appetite | 13 | 7 (4-7; 12) |
|  | Rash (all over) | 16 | 1 (1-2; 5) | Fever | 8 | 4 (3-7; 9) |
|  | Boil | 5 | 5 (3-7; 10) | Night sweats | 8 | 4 (3-7; 7) |
|  | Shingles | 3 | 5 (4-13; 18) | Chills | 6 | 3 (2-6; 13) |
|  | Mastitis | 1 | 14 (14-14; 14) | Migraine | 1 | 3 (3-3; 3) |
| Mouth/Dental  (123) | Mouth ulcer | 69 | 4 (2-6; 10) | Headache | 26 | 2 (2-4; 8) |
|  | Toothache | 45 | 3 (2-6; 10) | Muscle aches | 26 | 2 (1-6; 11) |
|  | Gum abscess | 24 | 6 (4-7; 10) | Fatigue | 23 | 5 (2-7; 14) |
|  |  |  |  | Loss of appetite | 9 | 4 (3-7; 14) |
|  |  |  |  | Chills | 7 | 3 (1-3; 5) |
|  |  |  |  | Night sweats | 6 | 7 (4-12; 18) |
|  |  |  |  | Fever | 5 | 3 (3-7; 11) |
|  |  |  |  | Migraine | 5 | 1 (1-2; 2) |
| Eye  (65) | Conjunctivitis | 36 | 4 (3-6; 7) | Fatigue | 33 | 5 (3-7; 27) |
|  | Red eye | 19 | 3 (3-5; 7) | Headache | 31 | 2 (1-5; 7) |
|  | Stye | 17 | 4 (1-7; 9) | Muscle aches | 28 | 3 (2-5; 25) |
|  |  |  |  | Loss of appetite | 17 | 5 (3-7; 15) |
|  |  |  |  | Night sweats | 12 | 4 (2-11; 40) |
|  |  |  |  | Fever | 11 | 3 (2-5; 7) |
|  |  |  |  | Chills | 8 | 2 (1-2; 4) |
|  |  |  |  | Migraine | 3 | 1 (1-2; 3) |

## Estimation of symptom duration

This analysis included reports from 559 participants on 1,281 infection syndromes (excluding syndromes treated with antibiotics). This data exhibits right-censoring: the date that symptoms resolved was not known for 133/1,281 (10%) syndromes, because follow-up ended before symptom reporting stopped. This would not be accounted for in empirical estimates of mean or median duration. We therefore opted for a model based approach. A range of parametric survival time models were estimated using the R survival package^1,2^: proportional hazard models (exponential, Gaussian, Weibull), and accelerated failure time models (lognormal, logistic, loglogistic). We selected the best-fitting model on the basis of the Akaike information criterion (AIC). The best-fitting model was loglogistic for every type of infection syndrome (Table S7). The duration median, interquartile range, and mean were computed from the model parameter estimates. Interval estimators for the mean are derived from asymptotic parametric approximations.^3^ They were computed using the R ciTools package.^4^ For respiratory tract infection syndromes, we estimated duration by age group (Table S8), and provided overall age standardised values (Table 3). We did not stratify by age group for other infection syndromes due to the smaller sample sizes.

We excluded syndromes for which antibiotic treatment was reported in this analysis. This was because of uncertainty in the effect that the antibiotic would have had on symptom length. However, we acknowledge that this may have also led to exclusion of some more severe syndromes.

## Table S7: Symptom duration model fitting by model and infection syndrome

| **Infection syndrome** | **Number of syndromes***  **(Total 1,281)** | **Number censored** | **Model Akaike Information Criterion (AIC)** | | | | | |
| --- | --- | --- | --- | --- | --- | --- | --- | --- |
|  |  |  | **Exponential** | **Weibull** | **Lognormal** | **Loglogistic** | **Logistic** | **Gaussian** |
| Respiratory | 757 | 87 | 4555 | 4556 | 4366 | 4368 | 5097 | 5605 |
| Gastrointestinal | 220 | 16 | 1296 | 1231 | 1133 | 1126 | 1624 | 1881 |
| Mouth/Dental | 111 | 6 | 699 | 699 | 659 | 655 | 799 | 925 |
| Skin/Soft tissue | 86 | 10 | 546 | 535 | 509 | 508 | 660 | 706 |
| Eye | 56 | 10 | 357 | 351 | 332 | 330 | 430 | 469 |
| Urinary tract | 51 | 4 | 271 | 272 | 253 | 251 | 319 | 347 |

*Number of syndromes used to estimate duration (excludes syndromes treated with antibiotics)

## Table S8: Age-specific estimates of symptom duration for respiratory infection syndromes

| **Age group (years)** | **Number of syndromes***  **(Total 757)** | **Crude** | | **Adjusted for right-censoring** | |
| --- | --- | --- | --- | --- | --- |
|  |  | **Median (IQR) days duration** | **Mean (sd) days duration** | **Median (IQR) days duration** | **Mean (95% CI) days duration** |
| 5 or less | 38 | 7 (4-12) | 10·4 (12·7) | 8·5 (4·8-15·0) | 13·9 (10·2-19·0) |
| 6-16 | 56 | 4 (3-6) | 5·0 (4·6) | 4·2 (2·4-7·5) | 6·9 (5·5-8·8) |
| 17-35 | 60 | 4 (3-7) | 6·9 (8·2) | 5·3 (3·0-9·4) | 8·7 (6·8-11·1) |
| 36-55 | 182 | 6 (3-12) | 9·5 (11·1) | 6·6 (3·7-11·6) | 10·8 (9·2-12·6) |
| 56-70 | 323 | 6 (3-13) | 10·4 (15·5) | 7·1 (4·0-12·6) | 11·7 (10·3-13·3) |
| 71+ | 98 | 7 (4-13) | 11·9 (17·3) | 7·9 (4·5-14·0) | 13·0 (10·7-15·7) |

IQR, interquartile range; sd, standard deviation; CI, confidence interval

*Number of syndromes used to estimate duration (excludes syndromes treated with antibiotics)

## Estimation of impacts on health-related quality of life

To estimate impacts on adult health-related quality of life, we identified syndromes reported by adults that did not lead to antibiotic use. Syndromes that led to antibiotic use were excluded because it could not be determined to what extent an antibiotic may have impacted health-related quality of life.

The EQ-5D-3L instrument has two components; a descriptive system and a visual analogue scale (EQ VAS).^5^ The descriptive system measures health across five dimensions (mobility, self-care, usual activities, pain, and anxiety), using a three-level scale (no problems, some problems, extreme problems). This generates a five digit number that represents a participant’s health state. The EQ VAS asks the participant to record their self-rated health from 0 (“Worst imaginable health state”) to 100 (“Best imaginable health state”). This is implemented in REDCap using a vertical slider and produces a single number between 0 and 100. Adults completed EQ-5D-3L at baseline and each day that they reported symptoms.

We mapped the five digit descriptive values to an index value using a validated UK data set ranging from one (full health) to zero (dead).^6^ We used the UK time trade-off value set to generate the EQ-5D index values. Within each infection syndrome, we calculated the mean EQ-5D index and EQ VAS score. We also identified the worst reported day by each metric and calculated the mean day on which this occurred, and the mean values. To estimate quality-adjusted life day (QALD) loss, we subtracted the daily EQ-5D index score from baseline scores. We estimated the mean daily QALD loss, and multiplied this by our duration estimates to calculate the mean and median QALD loss per syndrome.

## Table S9: Health-related quality of life at baseline

| **Number of adults** | **EQ5D index, mean (sd)** | **EQ5D-3L-VAS, mean (sd)** |
| --- | --- | --- |
| *502** | *0·88 (0·18)* | *82·7 (15·1)* |

*Number of adults (aged 16 or older) who reported at least one infection syndrome that did not lead to antibiotic use

## Table S10: Health-related quality of life impacts by infection syndrome

| **Infection syndrome** | **Number of syndromes*** | **Number of days** | **EQ5D index, mean (sd)** | | **EQ5D-3L-VAS, mean (sd)** | |
| --- | --- | --- | --- | --- | --- | --- |
|  |  |  | **All days** | **Worst day in syndrome** | **All days** | **Worst day in syndrome** |
| Respiratory tract* | 663 | 5981 | 0·83 (0·21) | 0·73 (0·28) | 66·2 (20·0) | 56·1 (22·3) |
| Gastrointestinal | 188 | 1332 | 0·67 (0·27) | 0·58 (0·34) | 63·2 (21·1) | 50·1 (22·0) |
| Mouth/Dental | 102 | 921 | 0·74 (0·24) | 0·65 (0·31) | 57·4 (25·5) | 62·3 (25·5) |
| Skin/soft tissue | 77 | 843 | 0·75 (0·25) | 0·68 (0·30) | 71·1 (18·4) | 67·9 (22·5) |
| Eye | 52 | 684 | 0·72 (0·23) | 0·68 (0·33) | 73·5 (15·5) | 59·9 (20·6) |
| Urinary tract | 49 | 268 | 0·70 (0·27) | 0·62 (0·38) | 56·5 (23·9) | 54·1 (24·2) |

*Number of syndromes reported by adults (aged 16 or older) that did not lead to antibiotic use

## Table S11: Background frequency of symptoms related to novel coronavirus (COVID-19) in Bug Watch cohort by day and month

| **Month** | **Number of days with completed surveys** | **Days symptom reported, number (%)** | | | |
| --- | --- | --- | --- | --- | --- |
|  |  | **Cough** | **Shortness of breath** | **Fever** | **Cough or shortness of breath or fever** |
| Jan | 15670 | 911 (5.81) | 213 (1) | 157 (1) | 1013 (6.46) |
| Feb | 11799 | 464 (3.93) | 134 (0.68) | 80 (0.68) | 518 (4.39) |
| Mar | 14140 | 417 (2.95) | 102 (0.52) | 74 (0.52) | 490 (3.47) |
| Apr | 13816 | 394 (2.85) | 131 (0.42) | 58 (0.42) | 448 (3.24) |
| May | 14748 | 382 (2.59) | 129 (0.59) | 87 (0.59) | 461 (3.13) |
| Jun | 9065 | 257 (2.84) | 129 (0.33) | 30 (0.33) | 289 (3.19) |
| Jul | 13805 | 261 (1.89) | 98 (0.37) | 51 (0.37) | 312 (2.26) |
| Aug | 15039 | 266 (1.77) | 81 (0.35) | 53 (0.35) | 330 (2.19) |
| Sep | 15896 | 516 (3.25) | 91 (0.48) | 77 (0.48) | 593 (3.73) |
| Oct | 11943 | 617 (5.17) | 131 (0.59) | 70 (0.59) | 687 (5.75) |
| Nov | 14702 | 567 (3.86) | 111 (0.79) | 116 (0.79) | 662 (4.5) |
| Dec | 18987 | 1027 (5.41) | 235 (0.78) | 148 (0.78) | 1125 (5.93) |

## Figure S1: Identification of incident infection syndromes


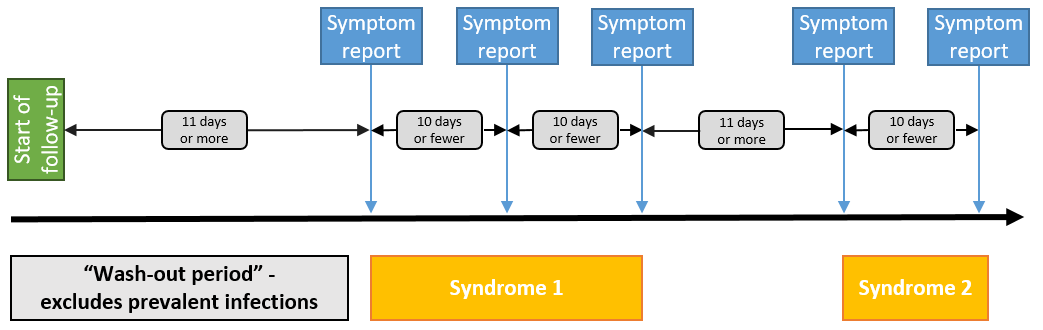


## Figure S2: Number of complete and incomplete weekly surveys


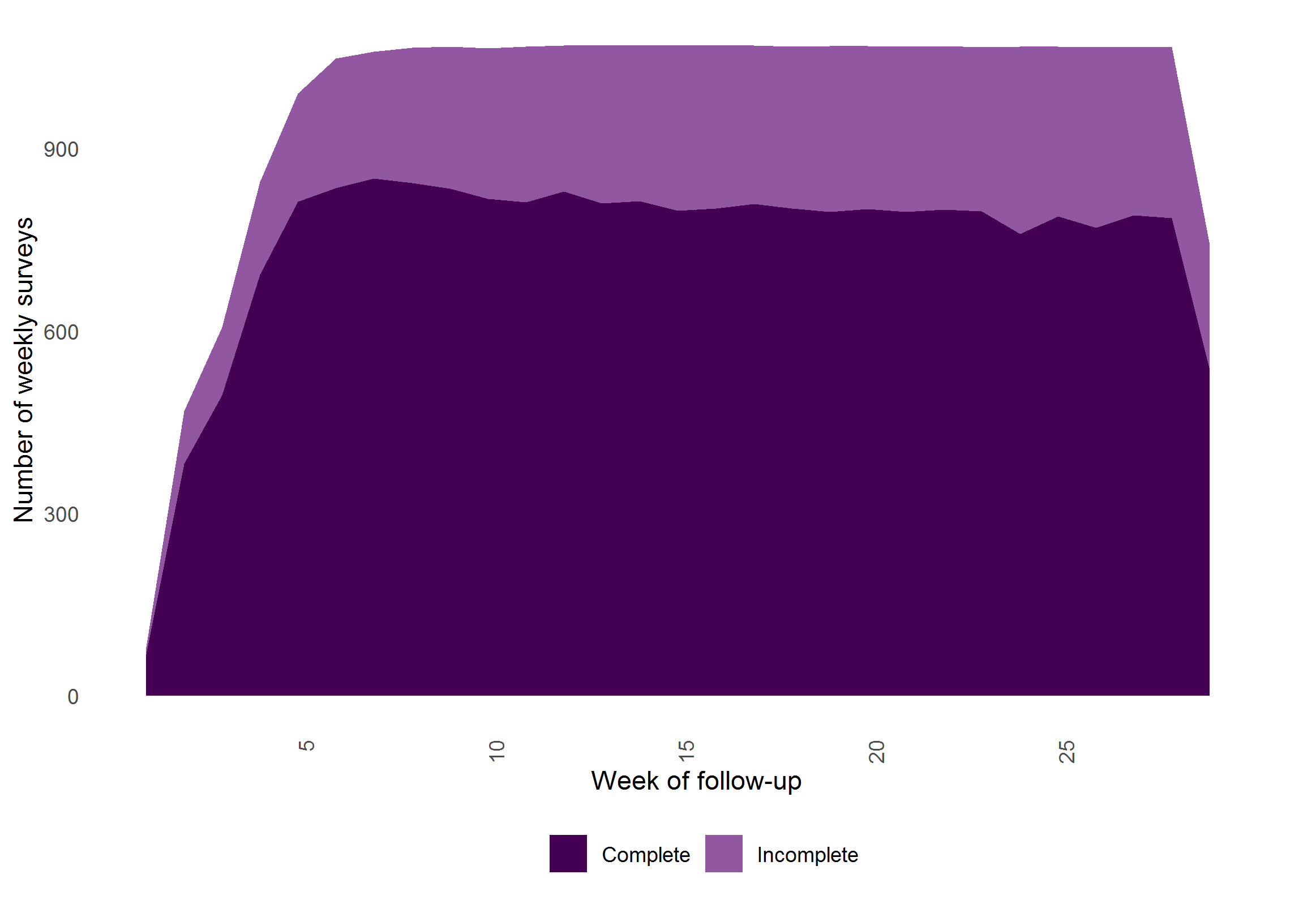


## Figure S3: Inclusion and exclusion of study participants


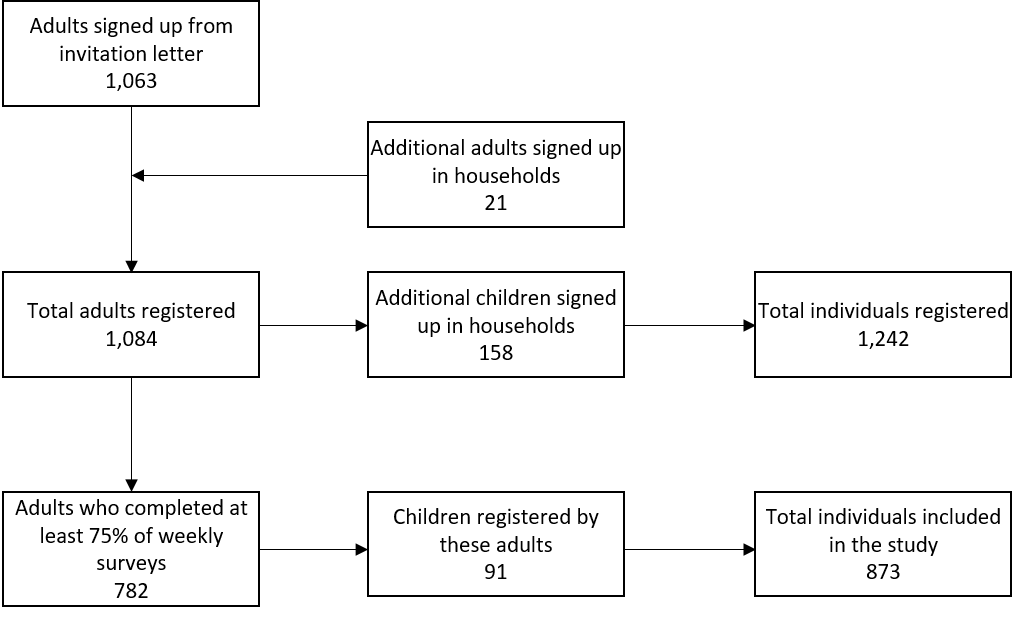


## Figure S4: Infection syndrome incidence by month


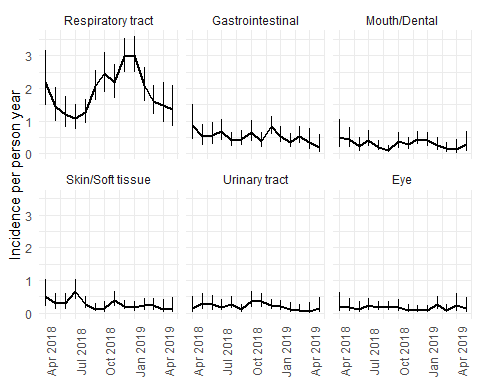


## Figure S5: Symptom reporting by infection syndrome


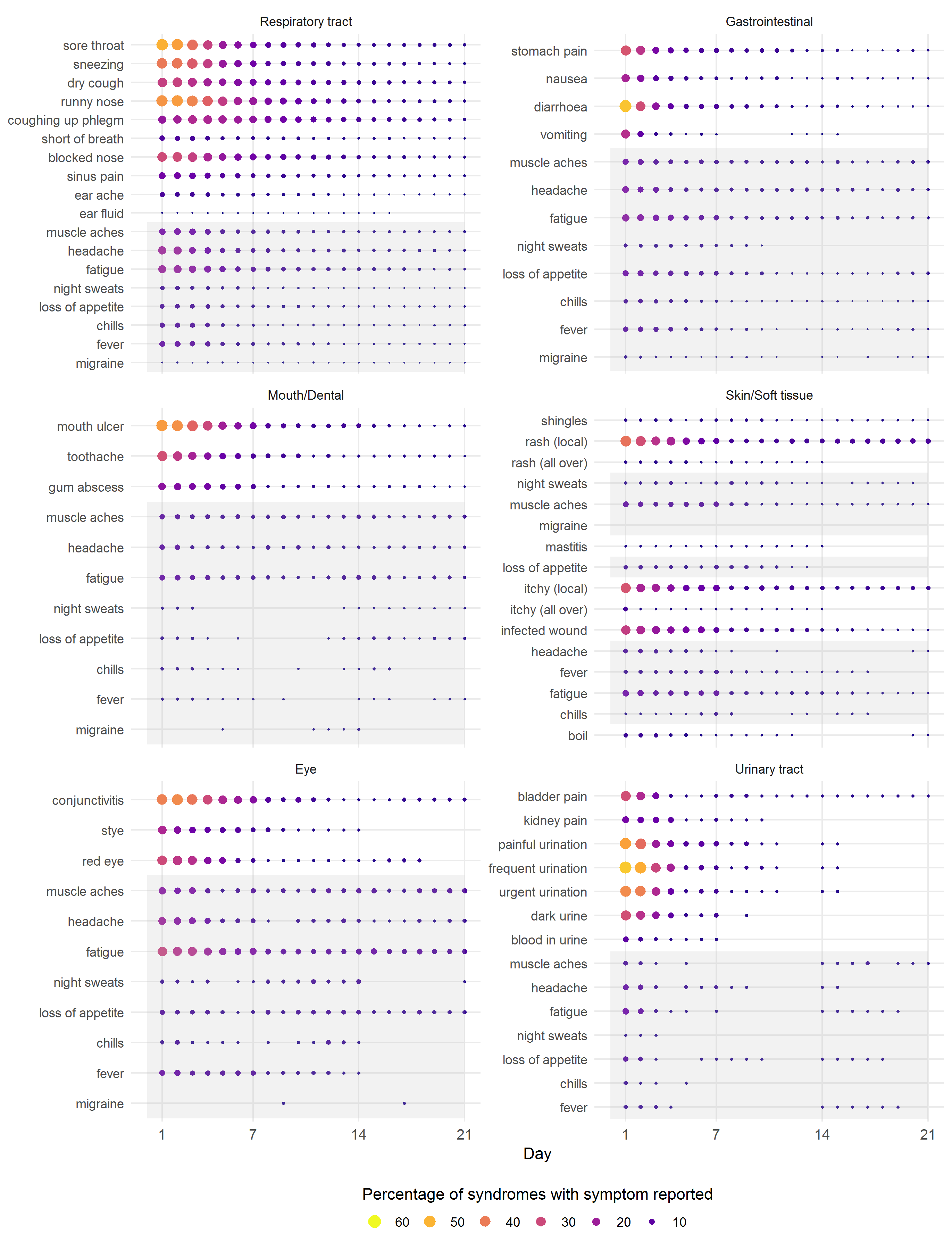


## References

1 Therneau T, Grambsch P. Modeling Survival Data: Extending the Cox Model. New York: Springer, 2000.

2 Therneau T. A Package for Survival Analysis in S. 2005.

3 Meeker W, Escobar L. Statistical methods for reliability data. John Wiley & Sons, 1998.

4 Haman J, Avery M. ciTools: Confidence or Prediction Intervals, Quantiles, and Probabilities for Statistical Models. 2019. https://cran.r-project.org/package=ciTools.

5 EuroQol Group. EuroQol--a new facility for the measurement of health-related quality of life. *Health Policy* 1990; **16**: 199–208.

6 Szende A, Janssen B, Cabases J. Self-Reported Population Health: An International Perspective based on EQ-5D. Dordrecht: Springer, 2014.
